# Owner Valuation of Rabies Vaccination of Dogs, Chad

**DOI:** 10.3201/eid1410.071490

**Published:** 2008-10

**Authors:** Salome Dürr, Martin I. Meltzer, Rolande Mindekem, Jakob Zinsstag

**Affiliations:** Swiss Tropical Institute, Basel, Switzerland (S. Dürr, J. Zinsstag); Centers for Disease Control and Prevention, Atlanta, Georgia, USA (M.I. Meltzer); Centre de Support en Santé International, N’Djaména, Chad (R. Mindekem)

**Keywords:** rabies, dogs, control, valuation, subsidies, Chad, Africa, willingness-to-pay, dispatch

## Abstract

We estimated the association between amount charged and probability that dog owners in N’Djaména, Chad, would have their dogs vaccinated against rabies. Owners would pay ≈400–700 CFA francs (US $0.78–$1.36)/animal. To vaccinate >70% of dogs, and thus interrupt rabies transmission, health officials should substantially subsidize these vaccinations.

Canine rabies globally causes an estimated 55,000 human deaths each year; 23,750 (≈43%) of which occur in Africa (*1*). To eliminate rabies virus in dog populations, and thus reduce the risk for human rabies, the World Health Organization (WHO) recommends dog rabies vaccination coverage of 70% (*2*). However, in most sub-Saharan countries, per capita expenditures on human health care are typically <$50/year (*3*), which makes securing funding to achieve the WHO target for dog vaccination challenging.

One way to fund dog rabies vaccination programs is to charge owners a fee for each dog vaccinated. However, the higher the fee, the lower the compliance is likely to be. To estimate the association between the amount charged to dog owners and the probability of vaccination (i.e., vaccination coverage), we collected data from 3 observational studies (2001, 2002, 2006) and 1 survey of dog owners (2006) (*4,7*; Table). We then estimated the maximum amount that could be charged to owners (cost recovery) and still achieve a minimum of 70% of dogs vaccinated.

## The Study

We used data collected in the capital of Chad, N’Djaména, which in 2001 had a human population of ≈776,000 and a dog population of ≈23,600 (*4*). Dog rabies is endemic to Chad; before the vaccination campaigns, the prevalence of dog rabies was ≈1.4–1.7 cases/1,000 unvaccinated dogs (*5*,*6*).

We obtained direct observations of the association between compliance (i.e., percentage of dogs vaccinated) and amount charged to owners from 2 pilot dog vaccination campaigns held in N’Djaména in 2002 and 2006 (*7*; unpub. data). Both campaigns followed similar protocols. Each campaign covered the same 3 city quarters, which had high-density dog populations (*4*). Only owned animals were vaccinated, but owned dogs comprise 90%–99% of all dogs in N’Djaména (*7*). Owners brought their animals (dogs, cats, monkeys) to 1 of 10 vaccination sites. In the 2002 campaign, vaccinations were free to owners (*7*); in the 2006 campaign, owners were charged 2,000 CFA francs (US $3.88)/animal vaccinated (unpub. data). (Exchange rate US $1 = 515.71 CFA francs as of February 2007; www.oanda.com/convert/classic) For each campaign, the percentage of dogs vaccinated was estimated by using a capture–recapture method (*7*; unpub. data). For the 2002 free-to-owners campaign, 71%–87% (95% confidence interval [CI] 64%–89%; mean 79%) of all dogs (owned and unowned) were vaccinated in 2 of the zones (1 zone per quarter) included in the campaign (*7*). For the 2006 campaign, in which owners were charged, the mean vaccination coverage among all dogs was estimated at 24% (95% CI 0.13%–24.82%) (unpub. data). Vaccination rates for owned dogs averaged only 78% and 25% in the 2002 and 2006 campaigns, respectively (*7*; unpub. data). For this study, we used the latter estimates because we were interested in measuring owner compliance to charges for dog vaccination.

Additional observational data were obtained from a household survey conducted in 2001 (*4*), which recorded that 19% of owned dogs were vaccinated against rabies. Such vaccinations would have been given at private clinics (i.e., without a campaign). The charge for such vaccinations at the urban government-run veterinary clinic and the 3 private veterinary practices of N’Djaména was 3,000–5,000 CFA francs (US $5.82–$9.69). We used the midpoint of such charges (i.e., 4,000 CFA francs). We did not inflate the 2001 charges because we encountered problems identifying an appropriate conversion factor that considered veterinary medical services.

During the 2006 campaign we surveyed dog owners by using a short questionnaire (Technical Appendix). The survey was conducted in the vaccination zones; households (containing at least 1 animal) were chosen randomly. The questions (written in French) were translated, when needed, into local languages by 4 interviewers. One question asked owners how much they were willing to pay for the vaccination of their animals.

We graphed the 3 observational data points (assuming a straight-line interpolation between points) and the reverse cumulative probability of the owner-stated amounts that they would be willing to pay for their animal to be vaccinated against rabies (Figure). An initial statistical (regression) analysis of the relationship between the amounts that owners said they would pay and the variables collected during the survey (Table) provided an adjusted *r*^2^ value of 0.07 (data not shown). We did not perform additional statistical analyses.

**Figure:**
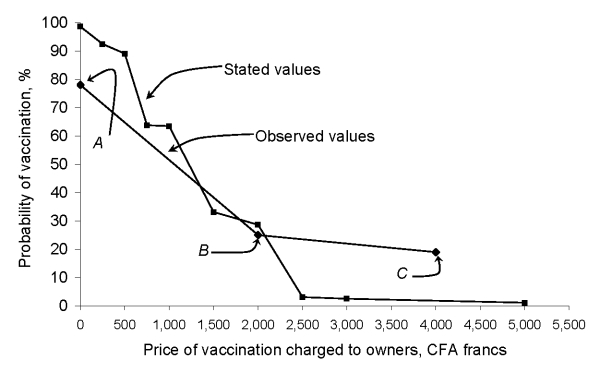
Average probability of having a dog vaccinated against rabies by charge for vaccination: observed versus owner-stated values for vaccination. The observed values of charges to vaccinate an owned dog against rabies and probability of vaccination came from 3 sources. Points A and B (recording vaccination coverage for all owned dogs vs. costs charged) come from 2 vaccination campaigns held in N’Djaména in 2002 and 2006, respectively. Point C represents the midpoint of the range of recorded 2001 clinic charges in N’Djaména for vaccinating a dog against rabies (costs not adjusted for any potential inflation). The owner-stated amounts that they would be willing to pay for their dogs to be vaccinated against rabies came from a survey of 356 households, conducted in 2006. The graph shows the reverse cumulative probability of the stated values.

**Table:** Characteristics of 356 persons interviewed and their households, N’Djaména, Chad, 2006

Characteristic	No. (%)
No. persons/household	
1–10	230 (65)
11–20	107 (30)
21–30	10 (3)
30–35	1 (0)
Unknown	8 (2)
Gender of persons interviewed*	
Female	165 (46)
Male	190 (53)
Unknown	1 (1)
No. animals/household	
Dogs	
0	7 (2)
1	278 (78)
2	65 (18)
3	6 (2)
Cats	
0	341 (96)
1	14 (4)
2	1 (0)
Monkeys	
0	346 (97)
1	10 (3)
Age of animals, y	
<1	101 (22)
1–<3	155 (34)
3–<6	134 (30)
>6	48 (11)
Unknown	12 (3)
Average	3.37
Sex of animals	
Male	346 (77)
Female	101 (22)
Unknown	3 (1)
Animals vaccinated >1 time	
Yes	314 (70)
Within past year†	197 (44)
Vaccinated during a campaign	121 (27)
No	132 (29)
Unknown	4 (1)

*Mean age of persons interviewed 33.7 y (range 13–80 y). †Confirmed by inspection of certificate of vaccination.

Interviewed households provided 356 questionnaires from which we estimated owner-stated willingness-to-pay for pet vaccination and calculated the resultant reverse cumulative probability of having their animal vaccinated. When asked how much they would be willing to pay, 5 (1%) owners stated that they were against vaccination. We interpreted that response to indicate that such owners would, essentially, have to be paid to have their animals vaccinated.

When the proposed cost of vaccination was <1,500 CFA francs/animal vaccinated, owners were more likely to state that they would pay to have their pet vaccinated than they were to actually do it. The stated values and observed values were closest at 2,000 CFA francs (≈25% probability of animal being vaccinated) (Figure). This finding was probably because the questionnaire was administered immediately after the campaign in which owners were charged 2,000 CFA francs/animal vaccinated. For >2,000 CFA francs, the observed values indicated that compliance would be greater than that stated by owners’ responses to the willingness-to-pay question. The Figure shows that to achieve a minimum of 70% of owned animals vaccinated, the maximum amount that could be charged would be ≈400 CFA francs (US $0.78) (observed values) to ≈700 CFA francs (US $1.36) (owner-stated values). Because the data shown in the Figure reflect owned animals only, to get vaccination coverage up to 70% of all animals (owned and stray), vaccination rates among owned animals would have to be >70%. To attain these higher rates, charges would have to be even lower than 400–700 CFA francs.

## Conclusions

Few studies have compared what members of the general public state they are willing to pay for a public health intervention with their actual observed behavior (*8*). Direct comparison between stated and observed behavior, as influenced by charges to owner, provides public health officials with an understanding of the reliability of owner surveys.

Our study and the data used have several limitations. First, the sample sizes were quite small (*6*,*7*). Furthermore, to maintain dog vaccination rates at the WHO-recommended rate of 70%, dog vaccination campaigns would have to be held every 1–6 years, which could reduce compliance. The survey was, by design, short, but a longer questionnaire may have allowed us to better identify why 75% of respondents did not wish to pay >500 CFA francs (US $0.97).

To achieve the WHO-recommended goal, public health officials cannot charge owners more than the equivalent of 400 CFA francs (US $0.78). Full-cost recovery concepts will not ensure that enough dogs are vaccinated in Chad (or, most likely, other African countries) to interrupt rabies transmission in dogs in urban areas. Clearly, to have >70% of all dogs vaccinated, public health officials and policy makers must consider methods to substantially subsidize dog rabies vaccinations.

## Supplementary Material

Technical Appendix Owner Valuation of Rabies Vaccination of Dogs, Chad
