# Supplementary material for: Owner Valuation of Rabies Vaccination of Dogs, Chad
Source: Emerg Infect Dis. 2008 Oct;14(10):1650–2. doi: 10.3201/eid1410.071490 (PMC2609875; doi:10.3201/eid1410.071490)
Supplement: Technical Appendix — Owner Valuation of Rabies Vaccination of Dogs, Chad [file 07-1490_Techapp-s1.pdf]

Owner Valuation of Rabies Vaccination of Dogs, Chad

Technical Appendix

Questionnaire for estimation of the vaccination coverage

Arrondissement: □ Quarter: ___________

Zone: □ Carré: □□

Name of investigator: ____________________

1. Consecutive number of the compound: □□□
2. Consecutive number of the household: □□□
3. Gender of interviewed person masculine □ feminine □
4. Age of the interviewed person |__|__| years
5. Number of persons in the household □□
6. In your household, how many do you have of dogs □ cats □ monkeys □

To fill out for each animal in the household:

7. Consecutive number of dog/cat/monkey (underline the according animal) □□

8. How old is your animal? □□ months

9. What is the gender of your animal male □ female □

10. Is your animal been vaccinated during the vaccination campaign? Yes □ 2. No □
(If not, go to the question number 13)

11. If yes, what is the colour of the collar given during the campaign? ________________

12. If yes (question 10), what symbol is painted on the animal's coat?
____
(Go to question 16)

13. If not, why didn't you brought the animal to the vaccination campaign?

---

14. Is your animal already vaccinated against rabies? yes □ no □
(If not, go to question number 17)

15. If yes, when is your animal been vaccinated? day □□ month □□ year □□

16. **Ask the vaccination certificate and control the date of the vaccination, note the vaccination's date:** day □□ month □□ year □□

17. How much are you ready to pay for the vaccination of your animal? __________FCFA

18. How many hours during the day your dog spend time outside of the compound?

0	0.5	1	2	3	4	5	6	7	8	9	10	11	12

19. Note whether the door is closed or not during the interview: yes □ no □

20. Note whether the dog is tethered yes □no □

21. After left the compound, note whether the dog is visible: yes □ no □
